# Infectious Complications in Metabolic and Bariatric Surgery: A Comprehensive Narrative Review of Pathophysiology, Prevention, and Management

**DOI:** 10.3390/life16050862

**Published:** 2026-05-21

**Authors:** Marcello Agosta, Egle Augello, Carlo Maria Bellanca, Andrea Marino, Cristiana Rossitto, Giuseppe Nunnari, Maria Sofia, Saverio Latteri

**Affiliations:** 1 Department of General Surgery, Cannizzaro Hospital, via Messina 829, 95126 Catania, Italy; cristianaros99@gmail.com (C.R.); mariasofia2002@libero.it (M.S.); saverio.latteri@unict.it (S.L.); 2 Department of General Surgery and Medical-Surgical Specialties, University of Catania, 95123 Catania, Italy; 3 Postgraduate Residency Program in Pediatrics, Department of Clinical and Experimental Medicine, University of Catania, 95123 Catania, Italy; 4 Department of Biomedical and Biotechnological Sciences, Section of Pharmacology, University of Catania, 95123 Catania, Italy; uni318437@studium.unict.it; 5 Department of Biomedical Sciences, Section of Neuroscience and Clinical Pharmacology, University of Cagliari, 09124 Cagliari, Italy; 6 Department of Clinical and Experimental Medicine, University of Catania, 95123 Catania, Italy; andrea.marino@unict.it (A.M.); giuseppe.nunnari1@unict.it (G.N.)

**Keywords:** bariatric surgery, metabolic surgery, surgical site infection, anastomotic leak, obesity, antibiotic prophylaxis, postoperative management, meta-inflammation

## Abstract

Background: Metabolic and bariatric surgery is an established therapeutic option for severe obesity and obesity-related medical problems. Although minimally invasive techniques and enhanced recovery pathways have reduced postoperative morbidity, infectious complications remain clinically relevant because they may lead to readmission, reoperation, prolonged antimicrobial therapy, and mortality. Methods: We conducted a narrative review of the literature on infectious complications after metabolic and bariatric surgery. Evidence was synthesized across five clinically relevant domains: host-related pathophysiology, microbial epidemiology, preoperative optimization, antimicrobial prophylaxis and pharmacokinetic considerations, and diagnosis and management of postoperative infectious complications. Results: Patients with obesity present specific infection-related vulnerabilities, including chronic low-grade inflammation, altered immune responses, impaired tissue oxygenation, obesity-related medical problems, and procedure-specific risks. Contemporary prevention relies on multidisciplinary preoperative optimization, appropriate skin antisepsis, weight-based antimicrobial prophylaxis, intraoperative redosing when indicated, and adherence to enhanced recovery principles. Anastomotic leaks and intra-abdominal abscesses represent the most severe organ/space infections and require early recognition, source control, antimicrobial therapy, nutritional support, and coordinated surgical, radiological, and endoscopic management. Conclusions: Infectious complications after metabolic and bariatric surgery result from the interaction between host physiology, microbial factors, pharmacological considerations, and surgical technique. A structured approach integrating prevention, early diagnosis, and multidisciplinary management may improve outcomes. Further bariatric-specific studies are needed to strengthen the evidence base for several preventive and therapeutic strategies.

## 1. Introduction

The global obesity epidemic has led to a significant increase in the number of metabolic and bariatric surgeries (MBS) being performed. Recognized not merely as a cosmetic intervention but as a critical metabolic therapy, MBS offers durable weight loss and the remission of associated medical problems such as type 2 diabetes mellitus (T2DM), hypertension, and obstructive sleep apnoea syndrome (OSAS) [1]. However, the benefits of these procedures are counterbalanced by the risk of perioperative complications, among which infections remain a predominant cause of morbidity, hospital re-admission, and mortality. The physiological milieu of the patient with obesity is characterised by chronic inflammation, altered immune competence, and unique pharmacokinetic challenges, creating a complex landscape for the prevention and management of infections.

Historically, the era of open bariatric surgery was plagued by high rates of surgical site infections (SSIs), driven by large incisions through poorly vascularized adipose tissue. The paradigm shift toward laparoscopic and, more recently, robotic techniques has drastically reduced the incidence of wound complications [2]. Nevertheless, the risk has not been eliminated. Modern bariatric surgery, while minimally invasive, involves complex gastrointestinal reconstructions that introduce the risk of organ/space infections, particularly anastomotic leaks and intra-abdominal abscesses. These complications often present insidiously, as the thick abdominal wall and blunted physiological reserves of patients with severe obesity can mask the classic signs of sepsis [3].

This narrative review aims to synthesize current evidence on the pathophysiology, prevention, diagnosis, and management of infectious complications after metabolic and bariatric surgery. Particular attention is given to host-related risk factors, microbial epidemiology, antimicrobial prophylaxis, surgical site infections, anastomotic leaks, intra-abdominal collections, and multidisciplinary postoperative management. The incidence of infectious complications varies significantly depending on the surgical approach, the specific procedure performed, and the patient’s baseline health status. Large-scale retrospective reviews report an overall rate of approximately 1% for infectious complications in laparoscopic bariatric surgery, which is significantly lower than the 10–15% rate often seen in open surgery. However, when the data is examined in more detail, a more nuanced picture emerges. SSI rates in laparoscopic cohorts range from 0.4% to 7.6%, while organ/space infections, such as anastomotic leaks, occur in 0.3% to 2% of Roux-en-Y gastric bypass (RYGB) cases and 1.5% to 3% of laparoscopic sleeve gastrectomy (LSG) cases [4].

While these percentages may appear low, the absolute number of affected patients is substantial when considered alongside the high volume of procedures performed globally. Moreover, the consequences of these infections are severe: indeed, postoperative infections are a leading cause of 30-day hospital readmission and are associated with a significant increase in mortality. For instance, pulmonary complications, including pneumonia, have been linked to an increased odds of 30-day mortality by almost 50-fold [5]. The burden is further compounded by the emergence of multidrug-resistant organisms (MDROs) and the unique challenges posed by viral pandemics (i.e., bariatric patients experienced a 156% increase in postoperative pneumonia rates during the SARS-CoV-2 pandemic) [6].

## 2. Materials and Methods

This article was designed as a narrative review. A literature search was conducted to identify relevant evidence on infectious complications after metabolic and bariatric surgery, with emphasis on pathophysiology, epidemiology, prevention, antimicrobial prophylaxis, diagnosis, and management.

Electronic searches were performed in PubMed/MEDLINE, Embase, the Cochrane Library, and Scopus from database inception to 13 April 2026. Search terms included combinations of Medical Subject Headings and free-text keywords related to “metabolic surgery,” “bariatric surgery,” “surgical site infection,” “anastomotic leak,” “intra-abdominal abscess,” “obesity,” “immune dysfunction,” “microbiota,” “antibiotic prophylaxis,” “cefazolin,” “pharmacokinetics,” and “postoperative infection.”

Eligible sources included randomized controlled trials, systematic reviews, meta-analyses, large observational studies, pharmacokinetic studies, and clinical guidelines from relevant scientific societies. Additional articles were identified through manual screening of reference lists when considered clinically relevant.

Exclusion criteria included articles not focused on adult patients, studies unrelated to metabolic or bariatric surgery or postoperative infections, non-clinical articles without translational relevance, duplicate publications, articles with insufficient methodological detail, and non-peer-reviewed sources when higher-quality evidence was available. Web-based sources and conference abstracts were avoided unless no peer-reviewed alternative was available or unless they provided specific society or institutional guidance.

Titles and abstracts were screened for relevance, followed by full-text assessment of selected articles. Evidence was organized thematically into five domains: host-related pathophysiological factors, microbial epidemiology and microbiota-related mechanisms, preoperative optimization, antimicrobial prophylaxis and pharmacokinetic considerations, and diagnosis and management of procedure-specific infectious complications. Because this was a narrative review rather than a systematic review, no formal meta-analysis or risk-of-bias assessment was performed. However, priority was given to recent systematic reviews, meta-analyses, clinical guidelines, randomized studies, and large observational cohorts. When evidence was indirect or extrapolated from general abdominal surgery or infectious disease literature, this was specified in the text.

## 3. Pathophysiological Features of Hosts with Obesity

Managing infections in the bariatric population effectively requires an understanding of the underlying host factors that predispose these patients to infection. Obesity is not a passive state of excess energy storage, but a chronic inflammatory condition that alters immune surveillance and wound healing. Adipose tissue, particularly visceral fat, has been shown to function as an active endocrine organ. Indeed, adipocytes secrete a milieu of pro-inflammatory cytokines, known as adipokines, including interleukin-6 (IL-6), tumour necrosis factor-α (TNFα), and leptin. These mechanisms become more pronounced the more severe the obesity, thus inducing a state of chronic, low-grade inflammation, often termed “meta-inflammation”, which in turn leads to a paradoxical suppression of the acute immune response, as suggested primarily by preclinical and observational data [7]. Furthermore, adipose tissue macrophages polarization shifts towards a pro-inflammatory M1 phenotype, which fuels chronic inflammation and is less effective at clearing acute bacterial pathogens compared to the anti-inflammatory M2 phenotype. This dysregulation is compounded by micronutrient deficiencies common in the bariatric population, such as vitamin D deficiency, which plays a crucial role in immune modulation and epithelial barrier integrity [7].

Neutrophils exhibit impaired chemotaxis and reduced oxidative burst capacity, which compromises their ability to eradicate invading bacteria at the surgical site [8]. Indeed, oxygen is a critical substrate for wound healing and resistance to infection. Nonetheless, adipose tissue itself is poorly vascularised, resulting in deficient oxygen-dependent killing mechanisms. Furthermore, as adipose tissue mass increases, the capillary density does not increase proportionally. This results in relative tissue hypoxia, which fosters a local environment favouring the growth of facultative and anaerobic organisms [9].

The physical biomechanics of obesity have also been demonstrated to contribute to tissue hypoxia. An increase in intra-abdominal pressure has been proven to have detrimental effects on venous return and lymphatic drainage, resulting in tissue oedema and further compromising microcirculatory flow. During surgery, the retraction of thick abdominal walls can cause localised pressure ischemia, further devitalising tissue and creating a nidus for infection. Research on tissue oxygen tension in obese patients following surgery has revealed that these patients frequently exhibit suboptimal levels of oxygen in the area surrounding the incision for extended periods of time. Observational studies have shown a direct correlation between reduced periincisional tissue oxygenation and an elevated risk of SSIs, although bariatric-specific data remain limited [9]. Bariatric surgery patients frequently exhibit pre-existing conditions, namely T2DM, OSAS, and cardiovascular diseases, which serve to amplify the risk of infection. Hyperglycaemia, even in the absence of a prior diagnosis of T2DM, has been demonstrated to exert a significant inhibitory effect on neutrophil function. It has been determined that perioperative hyperglycaemia (defined as a blood glucose level of >180 mg/dL) is an independent risk factor for SSIs, deep vein thrombosis, and mortality [7]. OSAS has been shown to contribute to intermittent hypoxia and systemic inflammation, in addition to increasing the risk of postoperative pulmonary complications, including pneumonia and atelectasis, which can serve as sources of secondary sepsis [5]. An additional and increasingly recognized dimension of obesity-related immune vulnerability is gut microbiota dysbiosis. In patients with severe obesity, the intestinal microbial community is typically characterized by reduced diversity, relative depletion of short-chain fatty acid (SCFA)-producing taxa such as *Faecalibacterium* and *Roseburia*, and expansion of pro-inflammatory Gram-negative species. This compositional shift is functionally relevant because SCFAs—particularly butyrate—are a primary energy source for colonocytes and a key regulator of intestinal epithelial tight-junction integrity. When SCFA production declines, the intestinal barrier becomes more permeable, facilitating translocation of bacterial endotoxins (lipopolysaccharide, LPS) into the portal and systemic circulation, a process often referred to as “metabolic endotoxaemia” [10,11]. The resulting chronic low-grade activation of Toll-like receptor 4 (TLR4) signalling amplifies the same pro-inflammatory cascade already driven by adipokines and M1-polarised macrophages, thereby reinforcing the meta-inflammatory state described above. In the perioperative context, dysbiosis-associated barrier dysfunction may increase susceptibility to bacterial translocation and organ/space infection following gastrointestinal reconstruction. Beyond pathophysiology, microbiota modulation is emerging as a potential therapeutic avenue. In other clinical settings, fecal microbiota transplantation (FMT) has shown the ability to restore microbial diversity and reduce systemic inflammatory markers; for example, a recent clinical trial in patients with alcohol-related cirrhosis demonstrated that FMT was associated with reduced hepatic encephalopathy severity and improvements in liver stiffness and steatosis, supporting a link between microbial restoration and attenuation of systemic inflammation [12]. Whether similar microbiota-targeted strategies can reduce postoperative infectious risk in bariatric patients remains to be determined and represents an important area for future investigation.

## 4. Microbial Landscape in Bariatric Surgery

Effective prevention and treatment of infections require an accurate understanding of the microbial landscape. The pathogens responsible for infections in bariatric surgery originate primarily from the patient’s endogenous flora. *Staphylococcus aureus* (including methicillin-resistant *S. aureus*) and coagulase-negative staphylococci (CoNS) are the predominant organisms causing superficial and deep incisional SSIs. The warm, moist skin folds of patients with severe obesity are often colonised by high microorganism loads, including *Candida* spp. and Gram-negative bacteria. Careful skin preparation is therefore necessary [8]. Organ/space infections (e.g., leaks and abscesses) are polymicrobial, involving both aerobic Gram-negative bacilli (e.g., *Escherichia coli* and *Klebsiella pneumoniae*) and anaerobes (e.g., *Bacteroides fragilis*). The specific flora encountered depends on the surgical site: proximal gastric procedures (such as sleeve gastrectomy and gastric bypass) involve upper gastrointestinal flora, which are less dense than colonic flora. However, the presence of reduced gastric acidity (due to the use of proton pump inhibitors or prior *Helicobacter pylori* gastritis) can facilitate bacterial overgrowth [13].

## 5. Discussion

### 5.1. Modifiable Risk Factors

The prevention of infectious complications begins before the patient reaches the operating room and depends on multidisciplinary optimisation of modifiable risk factors. Because perioperative hyperglycaemia is consistently associated with SSIs and other adverse postoperative outcomes, preoperative assessment should include haemoglobin A1c (HbA1c) to identify undiagnosed or sub-optimally controlled diabetes [14]. Although no single HbA1c threshold universally mandates delaying surgery, values ≥8–8.5% generally warrant optimisation before elective bariatric procedures. During the perioperative period, blood glucose should usually be maintained between 100 and 180 mg/dL, as tighter targets are not routinely recommended because they increase hypoglycaemia risk without clear additional benefit [15]. Accordingly, oral glucose-lowering agents are generally withheld on the day of surgery, SGLT2 inhibitors should be stopped at least 3 days beforehand, and insulin is the preferred agent when perioperative glycaemic management is required.

Many centres also use a short preoperative low-energy or very low-energy “liver-shrinking” diet, typically for 2–4 weeks. Its main purpose is to reduce liver volume and visceral adiposity, thereby facilitating laparoscopic exposure; it may also improve short-term glycaemic control and insulin resistance and, by simplifying the operation, may indirectly reduce infectious risk through shorter operative time, a recognised predictor of SSI. However, available evidence, derived mainly from systematic reviews of heterogeneous observational studies and small randomized trials, does not conclusively demonstrate that preoperative weight loss itself reduces postoperative complications [16,17].

Smoking is another major modifiable risk factor, as it impairs tissue oxygenation and healing and increases pulmonary, wound, marginal ulcer, and anastomotic complications. Current enhanced recovery after surgery (ERAS) and bariatric surgery guidance therefore support smoking cessation for at least 4 weeks before surgery, with longer abstinence likely conferring greater benefit; when abstinence is uncertain, biochemical verification such as cotinine testing may be considered, as self-reporting is not always reliable [18,19].

### 5.2. Skin Antisepsis

Preoperative skin antisepsis remains a key component of SSI prevention in bariatric surgery, although the current evidence no longer supports an unqualified statement that chlorhexidine is universally superior to povidone–iodine. Current SSI-prevention guidance recommends the use of an alcohol-based skin preparation, and the 2025 European update for gastrointestinal surgery specifically suggests alcohol-based chlorhexidine for clean, clean-contaminated, and contaminated fields when applied to intact skin rather than mucosal surfaces, as summarized in Table 1 [20]. Chlorhexidine may retain practical advantages because, unlike alcohol alone, it provides residual activity on the skin, a feature that may be relevant during longer procedures [21]. However, comparative evidence is more nuanced than earlier reviews suggested: an updated 2025 meta-analysis of randomized trials found lower overall SSI rates with chlorhexidine-based preparations, particularly in clean-contaminated surgery (RR 0.75, 95% CI 0.62–0.92), whereas a large 2024 randomized noninferiority trial in abdominal and cardiac surgery showed that povidone–iodine in alcohol was noninferior to chlorhexidine in alcohol [22,23]. Accordingly, in bariatric surgery, alcohol-based chlorhexidine can still be considered the preferred option when feasible, but alcohol-based povidone–iodine remains an acceptable alternative when chlorhexidine is contraindicated or unsuitable [20].

**Table 1:** Comparison of Skin Antiseptics for SSI Prevention.

Feature	Chlorhexidine Gluconate in Alcohol	Povidone–Iodine in Alcohol	Clinical Implication
Mechanism	Disrupts cell membranes and binds to the stratum corneum	Oxidation of cellular components	Both are effective antiseptics when used in alcohol-based preparations
Durability	Provides residual activity on the skin	Shorter residual activity	CHX may be advantageous during longer procedures
Comparative efficacy	Some meta-analyses suggest lower SSI rates, particularly in clean-contaminated surgery	A large randomized trial showed noninferiority compared with CHX in alcohol	Alcohol-based CHX may be preferred when feasible, but alcohol-based PVI remains an acceptable alternative
Practical considerations	Avoid on mucosal surfaces and when contraindicated	Useful alternative when CHX is contraindicated	Choice should consider surgical site, contraindications, and institutional protocols

### 5.3. Antibiotic Dosing Challenges in Bariatric Surgery

In patients with obesity, antimicrobial pharmacokinetics are influenced less by body weight per se than by the complex physiological changes accompanying excess adiposity. Obesity is associated with expansion of adipose tissue, a smaller and non-linear increase in lean mass and extracellular fluid, altered tissue perfusion, and variable changes in kidney and hepatic function, including augmented renal clearance in some patients and obesity-related liver dysfunction in others; together, these factors may modify both volume of distribution and drug clearance [24].

Drug distribution is often the pharmacokinetic phase most affected: lipophilic agents may show a greater increase in volume of distribution because of wider partitioning into adipose tissue, whereas hydrophilic antibiotics distribute mainly into lean body mass and extracellular water, compartments that increase to a lesser extent than total body weight [25]. However, these alterations are highly drug-specific and cannot be reliably inferred from total body weight alone. Consistent with the most recent systematic review and consensus guidance, obesity appears to cause only modest pharmacokinetic changes for most beta-lactams, and available evidence does not support routine dose escalation on the basis of obesity alone. Rather, dosing should be individualized according to the specific antimicrobial, renal function, site of infection, pathogen susceptibility, and, in the perioperative setting, the timing of administration and the need for redosing during prolonged procedures [26].

### 5.4. Antibiotic Prophylaxis Regimens

Building on these pharmacokinetic considerations, current practice supports a procedure-specific and weight-based prophylactic strategy rather than BMI-stratified dose escalation. For standard bariatric procedures involving the upper gastrointestinal tract, cefazolin remains the preferred first-line agent because it provides appropriate coverage against the organisms most commonly implicated in SSIs, as detailed in Table 2. In adults, it should be administered within 60 min before incision, using 2 g intravenously in most patients and 3 g in those weighing at least 120 kg and/or BMI > 50. When the procedure is prolonged, redosing is recommended after 4 h, or earlier in the setting of major intraoperative blood loss. Importantly, although obesity may reduce antibiotic penetration into subcutaneous adipose tissue, the most recent pharmacokinetic review and consensus guidance indicate that obesity alone does not justify routine dose escalation beyond these standard weight-based prophylactic regimens, since adequate exposure is usually achieved with cefazolin when administration and redosing are appropriate [27,28]. In patients with a true severe β-lactam allergy, prophylaxis should still ensure both Gram-positive and Gram-negative coverage, most commonly with clindamycin or vancomycin combined with an aminoglycoside, aztreonam, or a fluoroquinolone. Vancomycin should be started within 120 min before incision because of its longer infusion time and is best reserved for patients with documented MRSA colonization or other clear epidemiologic indications rather than used routinely. For conventional bariatric surgery, broader anaerobic coverage is not generally required and should be considered only when the procedure or intraoperative findings suggest more distal bowel involvement or contamination [29].

From a technical perspective, contemporary bariatric surgery should be framed within a broader SSI-prevention strategy rather than as an isolated question of antibiotic choice. Current ERAS recommendations favour a laparoscopic approach whenever feasible, and they do not support the routine use of abdominal drains or nasogastric decompression. Likewise, updated SSI-prevention guidance does not support continuation of prophylactic antibiotics after skin closure, even when a drain is left in place. Thus, in modern bariatric practice, effective infection prevention relies on the combination of timely pre-incision prophylaxis, appropriate intraoperative redosing, minimally invasive technique, and selective rather than routine use of drains or expanded antimicrobial coverage [30].

**Table 2:** Antibiotic Prophylaxis Guidelines in Bariatric Surgery.

Parameter	Recommendation	Evidence/Rationale
First-line prophylaxis	Cefazolin	Appropriate coverage for common Gram-positive organisms implicated in incisional SSI
Standard adult dose	Cefazolin 2 g IV within 60 min before incision	Recommended for most adult patients
Higher weight-based dose	Cefazolin 3 g IV for patients ≥120 kg	Weight-based adjustment recommended by current prophylaxis guidance
Redosing	Repeat cefazolin after 4 h, or earlier with major blood loss	Maintains adequate intraoperative concentrations during prolonged procedures
Severe β-lactam allergy	Clindamycin or vancomycin plus Gram-negative coverage, according to local policy	Ensures Gram-positive and Gram-negative coverage when cefazolin cannot be used
Anaerobic coverage	Not routine for standard upper gastrointestinal bariatric procedures; consider when distal bowel involvement or contamination is expected	Avoids unnecessary broad-spectrum prophylaxis

### 5.5. Incisional Surgical Site Infection

The Centers for Disease Control and Prevention (CDC)/National Healthcare Safety Network (NHSN) classification remains the most appropriate framework for bariatric surgery because it distinguishes superficial incisional SSI, confined to the skin and subcutaneous tissue, from deep incisional SSI involving fascia and muscle, and from organ/space SSI involving tissues deeper than the fascial plane. Notably, when more than one tissue level is involved, the event should be classified according to the deepest infected compartment. In laparoscopic bariatric procedures, trocar wounds are considered surgical incisions; therefore, port-site infections fall within the incisional SSI framework rather than representing a separate entity [31].

This distinction is clinically relevant because treatment intensity increases with depth. Superficial incisional infections are usually managed with local drainage and wound care, whereas deep incisional SSI generally requires operative debridement and systemic antimicrobial therapy. In patients with obesity, the local inflammatory picture may be less obvious because of body habitus, so persistent pain, progressive erythema, systemic toxicity, or failure of an apparently minor trocar-site infection to improve should prompt early reassessment for deeper involvement [32,33].

### 5.6. Organ/Space Infection: Leaks and Intra-Abdominal Collections

Whereas incisional SSIs are located at or near the surgical wound (skin, subcutaneous tissue, or fascia/muscle), organ/space SSIs involve anatomical structures that were opened or manipulated during the procedure but lie deeper than the fascial plane. In bariatric surgery, the most clinically important organ/space infection is a staple-line or anastomotic leak, because it combines luminal contamination with an intra-abdominal inflammatory response that may evolve rapidly from a contained collection to generalized peritonitis. The distinction is clinically relevant not only for classification but also for management: incisional SSIs are typically managed with local wound measures and, when indicated, systemic antibiotics and debridement (as discussed in Section 5.5), whereas organ/space infections usually require a source-control strategy integrating imaging, drainage, endoscopic therapy, and sometimes reoperation, as outlined below. Recent review-level data suggest that leaks after Roux-en-Y gastric bypass occur in approximately 1% to 2% of cases, whereas sleeve leaks are usually reported in the 0.8% to 4% range and most commonly arise near the angle of His. Their presentation may be deceptively subtle, and persistent or otherwise unexplained tachycardia should be regarded as an early warning sign, particularly when associated with fever, tachypnoea, oliguria, shoulder-tip pain, or rising inflammatory markers, as shown in Table 3. Computerised Tomography (CT) with both oral and intravenous contrast is the preferred first-line investigation because it improves visualization of both intraluminal anatomy and extraluminal collections; however, false-negative studies do occur, so clinical suspicion must remain paramount [34,35].

**Table 3:** Diagnostic Features of Anastomotic Leak.

Diagnostic Modality	Key Findings	Sensitivity/Specificity	Notes
Clinical Exam	Tachycardia (>120 bpm), Fever, Left Shoulder Pain	High Sensitivity (Tachycardia), Low Specificity	Tachycardia is the earliest and most reliable sign [36].
CT Scan (Oral Contrast)	Extravasation of contrast, Air/Fluid bubbles near staple line.	Sensitivity: ~60–80%/Specificity: >95%	Water-soluble oral contrast is generally preferred when leak is suspected. Negative CT does not rule out leak [37].
Upper GI Fluoroscopy	Contrast leak.	Sensitivity: Lower than CT.	Useful for functional assessment but misses small leaks [8].
Exploratory Surgery	Direct visualization of bile/food/pus.	Definitive diagnostic reference method in unstable patients or when clinical suspicion remains high despite negative imaging	Indicated for unstable patients or high suspicion despite negative imaging [38].

Management should be guided primarily by hemodynamic stability and by the adequacy of source control. The American Gastroenterological Association (AGA) Clinical Practice Update emphasises that endoscopic and other non-operative approaches should be reserved for selected hemodynamically stable patients and undertaken in a multidisciplinary setting with bariatric surgeons and interventional radiologists [39]. Accordingly, unstable patients or those with generalized peritonitis generally require urgent operative source control, whereas stable patients with contained leaks or localized collections can often be managed with antibiotics, nutritional support, image-guided or endoscopic ultrasonography-guided drainage when needed, and endoscopic therapy as shown in Table 4. Contemporary endoscopic management is no longer limited to covered stents; internal drainage with double-pigtail stents and endoscopic vacuum therapy are also established options [34,40]. Recent meta-analytic data show high overall success but substantial heterogeneity, indicating that treatment should be individualized according to leak timing, cavity characteristics, distal stenosis, and local expertise rather than following a single universal algorithm [41].

**Table 4:** Procedure-Specific Infectious Risks.

Procedure	Primary Leak Site	Leak Characteristics	Management Nuance
Sleeve Gastrectomy (LSG)	Angle of His (Proximal Staple Line)	High-pressure, ischemic.	Difficult to close; high risk of chronic fistula. Stents/Endo-Vac often used.
Roux-en-Y Gastric Bypass (RYGB)	Gastrojejunal (GJ) Anastomosis	Low-pressure.	More amenable to primary repair or endoscopic sealing.
One-Anastomosis Gastric Bypass (OAGB)	Gastrojejunal Anastomosis	Bile-rich, high volume.	Causes severe chemical peritonitis. Often requires conversion to RYGB.
Gastric Band (LAGB)	Port Site/Gastric Wall	Device infection/Erosion.	Late complication. Requires device explantation.

Intra-abdominal abscesses should be interpreted within the same source-control framework, as they frequently represent a contained leak or infected postoperative collection. CT remains the key diagnostic method, and when a collection is accessible, percutaneous drainage is generally preferred as part of source control, with antimicrobial therapy subsequently tailored to culture data and clinical response [42].

### 5.7. Other Postoperative Infections and Device-Related Issues

Not all infectious complications after bariatric surgery are SSIs. Pulmonary and urinary infections are strongly influenced by perioperative pathway adherence and pre-existing conditions, particularly OSAS and chronic respiratory disease. Current enhanced recovery after bariatric surgery (ERABS) recommendations support avoidance of routine nasogastric tubes, abdominal drains, and bladder catheters. They also support early mobilization and oral refeeding, and perioperative continuous positive airway pressure/non-invasive ventilation in appropriately selected patients with moderate-to-severe OSA. These measures shorten recovery and improve postoperative function without increasing major complications, therefore representing an important part of contemporary infection prevention after bariatric surgery [19].

When complications require central venous access for parenteral nutrition or prolonged treatment, standard central line-associated bloodstream infection prevention principles become especially important, as obesity itself is a recognized risk factor. Current international guidance emphasizes bundle-based insertion and maintenance practices together with daily review of ongoing line necessity. In the shrinking population of patients with previous adjustable gastric bands, recurrent late port-site infection should also raise suspicion of band erosion rather than being treated as an isolated superficial wound problem. In such cases, definitive management usually requires device removal, with endoscopic treatment preferred when technically feasible [43].

### 5.8. Integrative Synthesis and Knowledge Gaps

The preceding sections highlight that infectious complications after metabolic and bariatric surgery do not result from any single causative factor but from the convergence of host vulnerability, microbial ecology, pharmacological variability, and surgical technique. The chronic meta-inflammatory state and dysbiosis-related barrier dysfunction described in Section 3 and Section 4 create a baseline susceptibility that is further modulated by modifiable exposures—glycaemic control, smoking, and nutritional status—addressed in Section 5.1. Preventive interventions at the time of surgery, namely evidence-based skin antisepsis and weight-adjusted antimicrobial prophylaxis with appropriate redosing, target the transition from colonisation to infection but must be understood within the wider context of enhanced recovery principles, which collectively reduce infectious risk by minimizing tissue trauma, avoiding unnecessary devices, and promoting early physiological recovery.

When prevention fails, the distinction between incisional and organ/space infections determines the management trajectory. While superficial and deep incisional SSIs are generally amenable to wound-directed therapy, organ/space infections—particularly anastomotic leaks—demand a source-control paradigm that integrates clinical vigilance (tachycardia as an early sentinel sign), advanced imaging, and a graduated therapeutic response ranging from antibiotics and percutaneous drainage to endoscopic intervention and reoperation. The expanding endoscopic armamentarium, including covered stents, double-pigtail internal drainage, and endoscopic vacuum therapy, has broadened the non-operative options for hemodynamically stable patients, but the evidence supporting individual techniques remains largely observational, with considerable heterogeneity in reported outcomes.

Several important knowledge gaps persist. First, most data on obesity-related immune dysfunction and dysbiosis-driven barrier impairment derive from preclinical models or observational studies in non-surgical populations; prospective bariatric cohorts directly linking these mechanisms to postoperative infectious outcomes are lacking. Second, antimicrobial pharmacokinetics in patients with severe obesity are incompletely characterized for many commonly used agents beyond cefazolin, and therapeutic drug monitoring strategies have not been validated in the bariatric perioperative setting. Third, while enhanced recovery protocols are widely adopted, their individual bundle components have rarely been evaluated independently for infection-specific endpoints. Fourth, comparative effectiveness data for the various endoscopic approaches to staple-line and anastomotic leaks are limited by small sample sizes, retrospective designs, and centre-specific expertise, precluding firm algorithmic recommendations. Finally, emerging interventions such as microbiota-targeted therapies (probiotics, synbiotics, fecal microbiota transplantation) have a biological rationale for reducing infectious susceptibility in this population but have not yet been evaluated in adequately powered bariatric-specific trials. Addressing these gaps will require multicentre prospective studies with standardised infection definitions and long-term follow-up.

## 6. Limitations

This review has several limitations. First, it was designed as a narrative review rather than a systematic review; therefore, although the literature search was structured, no formal PRISMA-based selection process, quantitative synthesis, or risk-of-bias assessment was performed. Second, the available evidence on infectious complications after metabolic and bariatric surgery is heterogeneous. Some recommendations are supported by bariatric-specific studies, whereas others are extrapolated from general abdominal surgery, surgical site infection prevention guidelines, pharmacokinetic studies, or expert consensus. Third, the quality of evidence varies across topics. Data on anastomotic leaks, intra-abdominal abscesses, and postoperative source control are often derived from observational cohorts, retrospective studies, or expert reviews, while evidence for skin antisepsis and antimicrobial prophylaxis includes broader surgical populations that may not fully reflect bariatric-specific risk. Fourth, several mechanistic aspects, including obesity-related immune dysfunction, metabolic inflammation, dysbiosis, and altered tissue oxygenation, are biologically plausible and clinically relevant but are not always directly linked to postoperative infectious outcomes in bariatric cohorts. Finally, rapidly evolving areas such as microbiota-based interventions, endoscopic leak management, and antimicrobial dosing in patients with severe obesity require further prospective and bariatric-specific investigation. These limitations should be considered when interpreting the conclusions of this review.

## 7. Conclusions

Infectious complications after bariatric surgery remain a formidable clinical challenge, driven by the complex interplay of the patient’s metabolic physiology, the microbial environment, and surgical technical factors. While the transition to laparoscopy and the adoption of ERAS protocols have significantly reduced the incidence of surgical site infections, organ/space infections like anastomotic leaks continue to carry a high burden of morbidity.

Success in this field requires a relentless focus on prevention: rigorous preoperative optimization of glycemia and smoking status, weight-based antibiotic dosing that respects the unique pharmacokinetics of obesity, and appropriate skin antisepsis. When complications do occur, the clinician must maintain a high index of suspicion—recognizing that tachycardia may be the only sign of a catastrophic leak—and employ a multidisciplinary management strategy that integrates advanced imaging, interventional radiology, endoscopy, and timely surgical intervention. As the global volume of metabolic surgery continues to expand, these evidence-based strategies will be paramount in ensuring safe, durable outcomes for this vulnerable patient population.

## Data Availability

No new data were created or analyzed in this study.

## Abbreviations

The following abbreviations are used in this manuscript:

MBS	Metabolic and Bariatric Surgery
SSI	Surgical Site Infection
LSG	Laparoscopic Sleeve Gastrectomy
RYGB	Roux-en-Y Gastric Bypass
OAGB	One-Anastomosis Gastric Bypass
OSAS	Obstructive Sleep Apnoea Syndrome
